# Basal forebrain parvalbumin neuron dysfunction links network oscillation deficits to hippocampal pathology in Alzheimer’s disease

**DOI:** 10.21203/rs.3.rs-10027900/v1

**Published:** 2026-06-26

**Authors:** Eunjin Hwang, Hyun Soo Shim, Seung Chan Kim, Min-Ho Nam, Seung Jae Hyeon, Hea-Jin Kim, Ji Eun Kim, Hyeok Ju Park, Jiwan Woo, Eun Mi Hwang, Thor Stein, Junghee Lee, Jee Hyun Choi, Hoon Ryu

**Affiliations:** Korea Institute of Science and Technology; Korea Institute of Science and Technology; Korea Institute of Science and Technology; Korea Institute of Science and Technology; Korea Institute of Science and Technology; Korea Institute of Science and Technology; Korea Institute of Science and Technology; Dongguk University; Korea Institute of Science and Technology; Korea Institute of Science and Technology; Boston University Chobanian Avedisian School of Medicine; Boston University Chobanian Avedisian School of Medicine; Korea Institute of Science and Technology; Korea Institute of Science and Technology

**Keywords:** Alzheimer’s disease, Basal forebrain, Parvalbumin neurons, Hippocampus, Neural oscillations, Synaptic plasticity

## Abstract

**Background:**

Basal forebrain (BF) degeneration is a key pathological feature of Alzheimer’s disease (AD) and is closely associated with cognitive decline. Although parvalbumin (PV)-expressing neurons are abundant in the BF and are important regulators of cortical network activity, their contribution to AD pathogenesis remains poorly understood.

**Methods:**

To investigate the role of BF-PV neurons in AD, we examined the effects of PV knockdown in the BF of APP/PS1 mice. Electrophysiological recordings, behavioral analyses, retrograde tracing, hippocampal transcriptome profiling, and integrative computational analyses, including meta-correlation analysis and artificial neural network (ANN) modeling, were performed to assess the impact of BF-PV loss on neural network activity, hippocampal pathology, and cognitive function.

**Results:**

BF-PV knockdown disrupted theta oscillations and theta–gamma coupling in the parietal cortex and impaired hippocampal synaptic activity and memory-related behaviors in mice. Retrograde tracing demonstrated that BF neuronal circuits project directly to the hippocampus. Transcriptome analysis revealed that BF-PV knockdown increased amyloidosis- and microvessel-associated gene signatures while reducing synaptic plasticity–related gene expression in the hippocampus. Furthermore, meta-correlation analyses and ANN modeling indicated that BF-PV dysfunction strongly predicts hippocampal pathology, disrupted EEG coupling, and behavioral abnormalities in AD mice.

**Conclusions:**

These findings identify BF-PV neuronal dysfunction as an important contributor to hippocampal pathology, neural network dysregulation, and cognitive impairment in AD, highlighting BF-PV neurons as a potential mechanistic link between BF degeneration and AD progression.

## INTRODUCTION

Parvalbumin (PV)-positive neurons in the basal forebrain (BF) represent a distinct neuronal population that sends long-range projections throughout the central nervous system[1]. Unlike most inhibitory interneurons, BF-PV neurons form synaptic connections with interneurons in their target regions[2], including the prefrontal cortex[3, 4] and hippocampus[5, 6]. While the functions of PV interneurons within the cortex and hippocampus have been extensively studied, the physiological roles of BF-PV neurons—which may critically modulate activity in their projection targets—remain poorly understood.

Fast-spiking PV interneurons in the cortex play a central role in the generation of gamma-band oscillations[7, 8] and contribute to perceptual processing[9] and attentional processing[10]. In the hippocampus, PV interneurons are essential for generating theta rhythms[11], as well as for coordinating theta–gamma coupling[12] and associated memory functions[13]. Previous studies have shown that BF-PV neurons regulate prefrontal gamma-band oscillations[14, 15] and contribute to the reorganization of cortical networks during sensory processing[15]. Consistent with this idea, auditory steady-state responses are also known to be modulated by attention and top-down task demands[16–18]. However, the potential contribution of BF-PV neurons to higher-order cognitive functions remains largely unexplored.

The BF has long been implicated in the regulation of cognitive function and in the pathogenesis of Alzheimer’s disease (AD). Degeneration of BF subregions correlates with degeneration of cortical areas in a projection-specific manner in patients with AD[19–21], and the pattern of cortical atrophy is closely associated with the clinical manifestations of AD and mild cognitive impairment[22–24]. Notably, recent neuroimaging studies have demonstrated that atrophy of the BF predicts subsequent atrophy of the entorhinal cortex, but not vice versa[25], suggesting that BF degeneration may precede damage in the entorhinal cortex and hippocampus during AD progression.

Traditionally, the cognitive deficits associated with BF degeneration have been attributed to the loss of cholinergic neurons. However, the neurochemical basis of cognitive impairment in early-stage AD remains incompletely understood[26, 27]. Although neuronal loss[28, 29] and reductions in choline acetyltransferase (ChAT) activity[30, 31] have been reported in the BF of patients with advanced AD, subsequent studies indicate that the total number of BF neurons is relatively preserved during early stages of the disease[32, 33]. Moreover, ChAT activity in the isocortex and hippocampus does not appear to be significantly reduced during early AD[26–34]. Consistent with these findings, studies of cholinergic innervation have shown that partial memory function can be preserved even when BF cholinergic neurons are dysfunctional[35, 36]. Together, these observations suggest that additional neuronal populations and neurochemical mechanisms—beyond cholinergic signaling—may contribute to the early pathogenesis of AD.

In the present study, we investigated whether dysfunction of BF-PV neurons contributes to the molecular, cellular, electrophysiological, and behavioral abnormalities observed in AD. First, we examined whether BF-PV neurons are altered in postmortem brains from patients with neuropathological and clinical AD (NPCAD) and severe AD compared with normal subjects. We then assessed how PV knockdown in the caudal BF affects cortical theta oscillations, hippocampal theta–gamma coupling, and prefrontal–hippocampal communication in an AD mouse model. To explore potential molecular mechanisms, we performed transcriptome analysis of hippocampal tissues to determine how BF-PV loss influences synaptic plasticity–related gene expression. Finally, we conducted integrative meta-correlation analyses and artificial neural network (ANN) modeling to examine how BF-PV dysfunction is associated with electrophysiological alterations, pathological features, and behavioral deficits in AD mice. Our findings identify BF-PV neurons as a previously underappreciated regulator of hippocampal network activity and reveal their dysfunction as a potential driver of early AD pathogenesis.

## MATERIALS AND METHODS

### Human postmortem tissue

Postmortem brain tissues were neuropathologically examined and collected from normal subjects and patients with AD according to procedures established previously by the Boston University’s Alzheimer’s Disease Research Center (BUADRC). The patients underwent annual cognitive evaluations using the National Alzheimer’s Disease Coordinating Center (NACC) Uniform Data Set (UDS) protocol[37]. Consent for brain donation and research participation was provided by the donor’s next of kin. Institutional review boards from the Boston University Medical Center approved brain donation, postmortem clinical record review, neuropathological evaluation, and clinical interviews with family members of the donors. The neuropathological diagnosis of AD was established by board-certified neuropathologists based on the National Institute of Aging Reagan criteria and included intermediate or high probability[38]. In patients with AD, Braak Stages III to IV and V to VI were classified as mild cognitive impairment AD (neuropathological and clinical AD: NPCAD) and severe AD, respectively. Detailed information on the postmortem brain tissues is provided in Supplementary Table 1.

### Immunohistochemistry (IHC)

#### Alkaline phosphatase staining:

Paraffin-embedded human postmortem brain tissues were sectioned in a coronal plane at 10-20μm. Endogenous alkaline phosphatase was blocked using 3% hydrogen peroxide in TBS. Sections were blocked with 2.5% normal horse serum (Vector Laboratories) before incubation for 24 h using a mouse monoclonal antibody against PV (1:200 dilution; Cat. # 235, Swant, Marly, Switzerland). After washing, sections were incubated with the ImmPRESS-AP anti-mouse IgG (alkaline phosphatase) polymer detection reagent (Vector Laboratories, Inc., Burlingame, CA, USA) for 30 min at 20 ~ 22°C. Colors were developed with a Vector Red alkaline phosphatase substrate kit. Slides were subsequently counterstained with hematoxylin (Vector Laboratories, Inc., Burlingame, CA, USA), and processed back to xylene through an increasing ethanol gradient [70%, 80%, and 95% (1×), and 100% (2×)], and then mounted.

#### DAB staining:

The sections were blocked using 5% BSA with 0.5% Triton X-100 in PBS solution for 1 h at room temperature. Brain sections were incubated with a mouse monoclonal antibody against PV (1:200 dilution; Cat. # 235, Swant, Marly, Switzerland) at 4°C overnight. After washing three times, the slides were processed with the Vector ABC Kit (Vector Laboratories, Inc., Burlingame, CA, USA). The immunoreactive signals were developed with the DAB chromogen (Thermo Fisher Scientific, Meridian, Rockford, IL, USA) and analyzed under a bright-field microscope.

### Animals

B6C3-Tg(APPswe,PSEN1dE9)85Dbo/Mmjax (RRID:MMRRC_034829-JAX) mice (male; older than 7 months) were used in this study. Mice were maintained on a 12:12 h light:dark cycle (lights on at 8 am) with ad libitum access to food and water. Animal studies were performed in compliance with animal protocols approved by the Institutional Animal Care and Use Committee of the Korea Institute of Science and Technology (KIST) (Approval number 2018–019).

### Construction of AAV vectors containing PV shRNA

For *in vivo* gene silencing, the validated mouse PV shRNA sequences were cloned into the pSicoR vector using the HpaI/XhoI sites (Cat. # 21907, Addgene, Cambridge, MA, USA) and subcloned into the pAAV-MCS vector (Stratagene) using the MluI/BglII sites. High-titer AAV vectors were produced in HEK293TN cells using a helper virus-free system. Briefly, the AAV vectors were produced after co-transfecting with equimolar amounts of a rep/cap/helper plasmid. After incubation for 72 h, the cells were lysed, treated with benzonase (Cat. #E1014, Sigma), and further purified using HiTrap heparin columns (Cat. #17-0460-01, GE Healthcare, Pittsburg, CA, USA). Amicon ultra-15 centrifugal filter units (Cat. # UFC9100, Millipore, Billerica, MA, USA) were used to concentrate the sample up to the final volume.

### Surgery

Mice were anesthetized with a ketamine–xylazine cocktail (120 and 6 mg/kg, respectively) and fixed to a stereotaxic apparatus (51730D, Stoelting Co., Wood Dale, IL, USA; or Model 900, David Kopf Instruments, Tujunga, CA, USA). Subsequently, 1μL of AAV-PV-shRNA-mCherry was injected into the bilateral BF areas (AP, 0.0 mm; ML, ± 1.6 mm; DV, 5.3 mm from bregma) using a nano injector (Cat.# 53311, Stoelting Co., Wood Dale, IL, USA). Four micro-screw electrodes were chronically implanted in the bilateral medial frontal cortex (AP, + 2.3 mm; and ML, ± 0.6 mm from bregma) and the bilateral parietal cortex (AP, −2.0 mm; and ML, ± 2.0 mm from bregma; superficial to dorsal hippocampus). Two micro-screws were implanted on the interparietal bone and used as reference and ground electrodes, respectively. After the surgery, mice were housed individually in cages and were allowed a 2-week recovery period.

### Retrograde tracing using fluorogold

Fluorogold (4%; Fluoro-Gold, Fluorochrome, Denver, CO, USA) was used as a retrograde axonal tracer for identifying neurons that projected from the BF to the MS and the hippocampus. Fluorogold was microinjected into the MS and the dorsal and ventral hippocampi of mice. Mice were anesthetized with a ketamine–xylazine cocktail (120 and 6 mg/kg, respectively) and fixed to a stereotaxic apparatus (51730D, Stoelting Co., Wood Dale, IL, USA; or Model 900, David Kopf Instruments, Tujunga, CA, USA). Fluorogold (4%; in a total volume of 0.5μl) in normal sterile saline (0.9%) was microinjected using a 1μl Hamilton syringe at a rate of 0.1μl/min into the MS (AP, + 1.0 mm; ML, 0 mm; DV, −4.8 mm from bregma), the dorsal hippocampus (AP, − 2.0 mm; ML, ± 2.0 mm; DV, −1.8 mm from bregma), and the ventral hippocampus (AP, − 3.0 mm; ML, ± 2.8 mm; DV, − 3.8 mm from bregma). After infusion of the fluorogold solution, the needle was left in place for 10 min before it was slowly retracted. The animals were allowed 7 recovery days after the surgery and prior to experimentation.

### Acquisition and analyses of forced-walking EEG

To examine the functionality of hippocampal theta oscillations, EEG signals were recorded while the mice were actively walking on a treadmill (speed, 5 cm/s; LE8708 Single lane treadmill, Panlab, Barcelona, Spain) for 10 min. Before the treadmill walking, mice were allowed at least 10 min of habituation on the halted treadmill. EEG signals were acquired with sampling rate at 2 kHz using a Synamps2 amplifier and a SCAN 4.5 data acquisition system (Neuroscan, Inc., El Paso, TX, USA), then band-pass filtered between 0.1 and 100 Hz. EEG signals were analyzed with a custom-written MATLAB program. Signals were first high-pass filtered with a cut-off frequency 0.5 Hz. From the 10-min period during which the mice were actively walking on the treadmill, a 1-min epoch with maximal theta band power was sampled to calculate EEG power and CFC. EEG power was computed in delta (1–4 Hz), theta (6–12 Hz), SG (20–50 Hz), FG (60–100 Hz), and high-frequency oscillation (HFO; 120–160 Hz) bands using Welch’s method (10-s window with 5-s overlap). The correlation between EEG signals was computed using Pearson’s correlation coefficient. CFC of SG, FG, and HFO bands to the theta rhythm was calculated using the algorithm developed by Tort et al[39]. EEG power, correlation, and CFC were also computed for the 10-min habituation period and the 1-min epoch with maximal theta band power on a standstill treadmill.

### Behavior tests

#### Tube test of social dominance

The tube test apparatus is a 30-cm-long transparent acrylic tube with an internal diameter of 3 cm. Before the experiment, mice were habituated to the tube test apparatus for 10 min. On the following day, two mice from different groups (WT-scr, N = 8; WT-shPV, N = 7; TG-scr, N = 5; TG-shPV, N = 7) were placed at opposite ends of the tube and released simultaneously, to interact in the middle of the tube. A mouse that retreated fully from the tube (a full retreat was determined by the retrieval of all four paws out of the tube) was recorded as the loser. In turn, the mouse that remained inside the tube was recorded as the winner, thus ending the match. The number of wins was recorded as a percentage of the total number of matches.

##### Nest-building test:

Nest material (Nestlet; Ancare; UK agent, Lillico, 5 cm × 5 cm, 3.0 g) was placed in each individual home cage (WT-scr, N = 8; WT-shPV, N = 12; TG-scr, N = 8; TG-shPV, N = 9). After 24 h, the nests were scored using a 6-point system: (0) Nestlet not touched, (1) Nestlet almost not torn (more than 90% intact), (2) Nestlet partially torn (50%–90% remaining intact), (3) Nestlet mostly torn but no identifiable nest site, (4) An identifiable but flat nest, (5) A perfect nest with a wall higher than the mouse’s height. This system was modified from the method reported by Deacon[40].

##### T-maze reversal learning:

Before the experiments, all subjects were habituated to a T-maze (entry arm, 35 × 10 cm; T-arms, 30 × 10 cm each) and learned to consume the sucrose pellets (Rodent Purified Diet, Bio-Serve, Flemington, NJ, USA) from the food cups placed at the end of each arm for 5 days. On the following day, the sucrose pellets were placed in a food cup in one of the T-arms (correct arm) and all subjects were trained to search for these sucrose pellets in 10 consecutive daily trials, until they reached a learning criterion: that the subject entered the correct arm and consumed the reward in at least 6 trials among 10 on 3 consecutive days. The correct arm was counterbalanced across subjects. The subject was allowed 3 min to complete each trial. After a subject reached the discrimination learning criterion, the correct arm was changed to the opposite T-arm and 10 daily trials were repeated until the criterion was reached again (reversal learning criteria) (WT-scr, N = 8; WT-shPV, N = 12; TG-scr, N = 8; TG-shPV, N = 9).

#### Open Field Test

The open field test was administered for 10 min in a white acrylic chamber (40 × 40 × 40 cm) under a light intensity of 4 lux. The center zone was defined as the central square (20 × 20 cm) located in the middle of the open field arena, and the EthoVision XT (Noldus, Wageningen, the Netherlands) software was used to track total distance, duration, and number of entries in the center zone (WT-scr, N = 8; WT-shPV, N = 12; TG-scr, N = 8; TG-shPV, N = 9).

#### Object/location novelty recognition test

On the day following the OFT, during the sample object phase, two identical sample objects were placed in the two corners of the box, and the mice were allowed to explore the arena and objects freely for 10 min. The novel object phase was scheduled for 24 h later, in which the mice were placed into the arena again, and one of the sample objects was replaced with a novel object. Mice were allowed to explore the arena for 5 min, and the time spent exploring each object was scored. Twenty-four hours later, during the NOL phase, one of the two objects that were used in the novel object phase was moved to a novel place in the arena, and the mice were tested in the same way as before. The discrimination index was calculated as the ratio of time spent exploring the novel object or the object that was moved to the novel location to the total time spent exploring the two objects.

### Immunofluorescence staining and confocal microscopy

The sections were blocked using 5% BSA with 0.5% Triton X-100 in PBS solution for 1 h at room temperature. Brain sections were incubated with a rabbit anti-BDNF monoclonal primary antibody (1:200; Cat. # ab1534, Millipore, Burlington, MA, USA), a rabbit anti-APP polyclonal primary antibody (1:500; Cat. # NBP2-15575, Novus, Centennial, CO, USA), a goat anti-ChAT monoclonal primary antibody (1:400; Cat. # a1440, Millipore, Burlington, MA, USA), a rabbit anti-NEFM monoclonal primary antibody (1:200; Cat. # ab64300, Abcam, Cambridge, MA, USA), or a chicken anti-mCherry antibody (1:1000; Cat. # ab205402, Abcam, Cambridge, MA, USA) at 4°C overnight. After three rinses with PBS, the sections were incubated with the secondary antibodies (FITC-conjugated donkey anti-rabbit, goat, or mouse IgG antibodies (1:1000) and Alexa fluor 594-conjugated donkey anti-chicken IgG antibodies (1:1000) (Jackson ImmunoResearch Laboratories, West Grove, PA, USA)) for 2 h at room temperature. Sections were then mounted and counterstained using gelatin-containing DAPI. Fluorescence was observed by confocal microscopy (Nikon A1R, JAPAN). Pre-absorption with an excess of the target protein or omission of the primary antibody were used to demonstrate antibody specificity and the background generated by the detection assay, respectively. Colocalization and quantitative assessment of the images were carried out using the NIH Image J software.

### Transcriptome (RNA) sequencing and analysis of mouse hippocampal tissues

Total RNA was isolated using the TRIzol reagent (Invitrogen), and RNA quality was assessed on an Agilent 2100 bioanalyzer using the RNA 6000 Nano Chip. RNA library construction was performed using a QuantSeq 3′ mRNA-Seq library prep kit (Lexogen, Inc., Austria). High-throughput sequencing was performed as single-end 75 sequencing using NextSeq 500 (Illumina, Inc., USA). QuantSeq 3′ mRNA-Seq reads were aligned using Bowtie2[41]. The normalized gene expression data were obtained based on a quantile normalization method using edgeR[42].

### Differential gene expression analysis in experimental mice

For the generation of the heatmap, the centered expression counts were applied to the average linkage clustering algorithm using Cluster 3.0[43], and the results were visualized with Java Treeview[44]. We defined DEGs as genes with |Foldchange| ≥ 1.5 and |experimental mouse normalized counts–control mouse normalized counts∣ ≥ 1. To identify pathways that were significantly enriched in DEGs, we used the gene ontology molecular function database from MSiqDB.

### Quantitative real-time PCR (qPCR)

qPCR was performed on the ABI PRISM 7700 Sequence Detection System Instrument and software (Applied Biosystems, Foster City, CA, USA) using the manufacturer-recommended conditions. Total RNA was isolated from transiently transfected cells (TRIzol reagent, Invitrogen, CA), reverse transcribed (Superscript III, Invitrogen, CA), and subjected to quantitative PCR analysis using the SYBR green master mix (Invitrogen, CA). The comparative threshold cycle (Ct) method was used to calculate the amplification factor, and the relative amount of targets was normalized to GAPDH levels in parallel reactions. The primer sequences are described in Supplementary Table 2.

### Hippocampal slice preparation

Two mice from each group were deeply anesthetized with isoflurane, followed by decapitation. The brains were removed from the skull and placed in ice-cold oxygenated (95% O_2_ and 5% CO_2_) artificial cerebrospinal fluid (ACSF; 130 NaCl, 24 NaHCO_3_, 3.5 KCl, 1.25 NaH_2_ PO_4_, 1 CaCl_2_, 3 MgCl_2_, and 10 glucose (in mM); pH 7.4). Transverse slices with a thickness of 300μm thickness were cut on a vibrating microtome (Dosaka, Pro-7N, Kyoto, Japan) and stored in an incubation chamber at room temperature for at least 1 h before recordings.

### Slice recording

Hippocampal slices were transferred to the recording chamber, which was constantly perfused with oxygenated ACSF composed of 130 NaCl, 24 NaHCO_3_, 3.5 KCl, 1.25 NaH_2_PO_4_, 1.5 CaCl_2_, 1.5 MgCl_2_, and 10 glucose (in mM) saturated with 95% O_2_ and 5% CO_2_, at pH 7.4. For voltage-clamp experiments, the solution used to fill the electrodes was composed of 140 CsMeSO_4_, 10 HEPES, 7 NaCl, 4 Mg-ATP, and 0.3 Na_3_-GTP (in mM). Visually guided whole-cell patch recordings were obtained from CA1 pyramidal neurons in the voltage clamp or current clamp configuration using a MultiClamp 700B amplifier (Molecular Devices, San Jose, CA, USA) and a borosilicate patch pipette with a resistance of 5–8 MΩ. All neurons included in this study had a resting membrane potential below – 55 mV and an access resistance in the range of 20–60 MΩ, and showed only minimal variation in these parameters during the recording period. Recordings were filtered at 2 kHz and digitized at 10 kHz using a Digidata 1322A instrument (Molecular Devices, San Jose, CA, USA). sEPSC and sIPSC recordings were started after initial stabilization of the baseline and were maintained for at least 5 min. sEPSCs and sIPSCs from the last 3-min recordings were included in the analyses.

### Meta-correlation analysis and network graphing

Data pertaining to a total of 218 features (181 EEG, 24 behavioral, 9 pathological, and 4 patch-clamp features) from 32 mice (WT-scr, n = 8; WT-shPV, n = 7; TG-scr, n = 8; TG-shPV, n = 9) were z-transformed. Spearman’s rank correlations between the pairs of features were computed and the null hypothesis of no correlation was tested for each pair. Partial correlation between pathological features was computed using the partialcorr function in MATLAB. Significant connections between the features were visualized using Gephi[45]. The alpha level for statistical significance was set at 0.05, unless otherwise noted.

### Circulogram

Given the set of group pairs $\left\{\left(g_{1}^{1},g_{1}^{2}\right),\cdots ,\left(g_{i}^{1},g_{i}^{2}\right),\cdots ,\left(g_{n}^{1},g_{n}^{2}\right)\right\}$, statistical patterns $\left\{c_{1},c_{2},\cdots ,c_{n}\right\}$ were defined as $c_{i}=-1$ if a significant difference between $g_{i}^{1}$ and $g_{i}^{2}$ was required, and $c_{i}=1$ otherwise. Subsequently, the statistical pattern of feature f was quantified using the n-dimensional significance vector ${\vec{s}}^{f}$, as follows.

$${\left({\vec{s}}^{f}\right)}_{i}=c_{i}∙H\left(0.05-p_{i}^{f}\right),$$

where $p_{i}^{f}$ is the P-value from the rank-sum test of f between $g_{i}^{1}$ and $g_{i}^{2}$ and H(x) is the Heaviside step function. From these significance vectors, the similarity of statistical behaviors between the two features $f_{i}$ and $f_{j}$ was defined as follows.

$$\text{similarity}\;\left(f_{i},f_{j}\right)={\vec{s}}^{f_{i}}\cdot {\vec{s}}^{f_{j}}.$$

Based on the calculated similarities and a threshold (k=2), feature nodes were connected if their similarities were larger than k.

### Construction of an ANN for predicting genotype and shPV knockdown

In this study, a total of 218 features (181 electrophysiological, 24 behavioral, 9 pathological, and 4 patch-clamp features) were obtained from 32 mice (WT + scr, n = 8; WT+shPV, n = 7; TG + scr, n = 8; TG+shPV, n = 9). Missing data were imputed based on the group mean of the corresponding feature, if any. All features were rescaled by z-transformation to obtain standardized coefficients. An ANN-based classifier was constructed using *layer_dense* function building multilayer perceptron (MLP) architectures in the Keras package in R (version 2.2.4.1, https://github.com/rstudio/keras/). The dataset was randomly divided into training and testing sets, while retaining the near 7:1 ratio in both classes. The model was trained with the ADAM optimizer, which is a first-order gradient-based optimization algorithm[46], for efficient and fast optimization. The accuracy was computed on 20 repetitions of stratified 8-fold cross-validation, and the hidden-layer size and unit were selected to maximize the accuracy. The candidate models were further trained up to 30 epochs with a batch size of 4 using the ADAM optimizer and cross-entropy loss (see **Supplementary Fig. 11B** for the loss and accuracy plot).

### Determination of high attribution features in the ANN

The contribution of individual features to the prediction of genotypes was determined based on gradient-based attribution methods for ANN[47]. Inspired by saliency maps that visualize the inputs of high attribution[48], we defined an attribution vector, ${\vec{d}}^{g}=\left[d_{1}^{g},\ldots ,d_{N}^{g}\right]\in R^{N}$ of N input features, $\vec{x}$ to the output, g according to the following steps. First, two unit vectors pointing in the direction of each genotype contrast, WT-TG and scr-shPV, were determined in a 2-D principal component (PC) plane constructed using the hidden units in the first layer of the ANN. Second, the magnitude of the projection over each unit vector was calculated for each hidden unit and then assigned as $k_{j}^{g}$ for the $j^{\text{th}}$ hidden unit. Next, the attribution of the $i^{\text{th}}$ feature for contrast g, $d_{i}^{g}$, was calculated by summating the absolute value of the partial derivative of the hidden unit with the $i^{\text{th}}$ feature, i.e., $\sum_{j}\frac{\partial k_{j}^{g}}{\partial x_{i}}$, to assess the level of change by the perturbation with the $i^{\text{th}}$ feature. Lastly, the overall attribution value of the $i^{\text{th}}$ feature, $D_{i}$, was calculated as $\sqrt{\sum_{g}{\left(d_{i}^{g}\right)}^{2}}$, and the features of the top 5% attribution were determined to be the high attribution features.

In greater detail, the hidden unit, $y_{j}$, in MLP was given as:

$$y_{j}=S\left(\sum_{i=1}^{N}W_{ji}x_{i}+b_{j}\right),$$

where S is a sigmoid function and N is the number of input features. Then, $y_{j}$ is clustered to be ${\vec{y}}^{g}$ for any group according to its predictability. Subsequently, the unit vector was determined by the clusters in the PC plane:

$${\hat{e}}^{TG}=\frac{{\vec{y}}^{TG}-{\vec{y}}^{WT}}{\left\|{\vec{y}}^{TG}-{\vec{y}}^{WT}\right\|}\text{and}\;{\hat{e}}^{WT}=-{\hat{e}}^{TG},\;\text{and}$$

$${\hat{e}}^{scr}=\frac{{\vec{y}}^{scr}-{\vec{y}}^{\text{shPV}}}{\left\|{\vec{y}}^{scr}-{\vec{y}}^{\text{shPV}}\right\|}\text{and}\;{\hat{e}}^{\text{shPV}}=-{\hat{e}}^{scr}.$$

### Statistics

Statistical analyses were performed using Prism 7 or MATLAB. Differences between two different groups were analyzed with the two-tailed Student’s unpaired t test or Mann-Whitney test (when the data is not normally distributed). For assessment of change of a group by a certain intervention, the significance of data was assessed by the two-tailed Student’s paired t test. For comparison of multiple groups, one-way analysis of variance (ANOVA) with Tukey’s or Dunnett’s multiple comparison test, or two-way ANOVA with Bonferroni’s multiple comparison test was assessed. Data from multiple independent experiments were assumed to follow a normal distribution. p<0.05 was considered to indicate statistical significance throughout the study. Unless otherwise specified, all data are presented as mean ± SEM. No statistical method was used to predetermine sample size. Sample sizes were determined empirically based on our previous experiences or the review of similar experiments in literature. The numbers of animals used are described in the corresponding Fig. legends or on each graph.

## RESULTS

### PV levels are reduced in the NBM of AD postmortem brains and in 5×FAD mice

**Figure 1:**
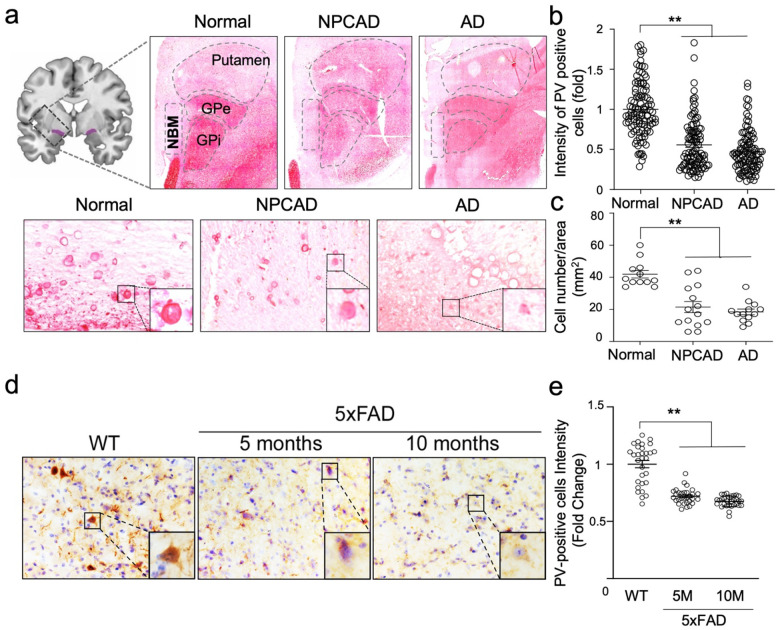
The PV levels of the NBM were reduced in human postmortem AD brains and in the 5×FAD mouse model of AD. **(a)** PV immunoreactivity was decreased in the NBM of AD (N = 5) and NPCAD (neuropathological and clinical AD, N = 5) postmortem brains compared with normal brains (Normal, N = 5). **(b)** The intensity of PV was significantly decreased in the NBM of AD and NPCAD brains. **(c)** The number of PV-positive cells was significantly decreased in the NBM of AD and NPCAD brains. **(d**) PV immunoreactivity was decreased in an age-dependent manner in the MCPO of brains from 5 (N = 3) and 10 month-old 5×FAD mice (N = 3) compared with WT mice (N = 3). **(e)** The intensity of PV was significantly decreased in the MCPO of brains from 5 and 10 month-old of 5×FAD mice. A total of 30 cells was counted (10 cells per mouse) in each group of mice (N = 3). Statistical significance was analyzed by two-way ANOVA followed by Tukey’s multiple-comparisons test. Data are presented as mean ± SEM. ** P<0.01.

BF degeneration is a hallmark of AD and is strongly associated with cognitive decline. Although cholinergic dysfunction in the nucleus basalis of Meynert (NBM) has been extensively studied, whether non-cholinergic neuronal populations in this region are also affected during AD progression remains unclear. Because parvalbumin (PV)-expressing neurons play critical roles in regulating cortical network activity, we first asked whether PV expression is altered in the NBM in AD. To address this question, we performed immunohistochemistry (IHC) for PV in the nucleus basalis of Meynert (NBM) of postmortem brains of normal subjects and patients with mild and severe AD. Anatomically, the main body of the NBM lies inferior to the anterior commissure and the globus pallidus, and lateral to the anterior hypothalamus, in an area known as the substantia innominate (Fig. 1a)[49]. Morphological analysis revealed that PV-positive neurons in the NBM were markedly shrunken in severe AD and NPCAD brains compared with those in normal controls. A densitometry analysis showed that PV immunoreactivity was significantly decreased in the NBM of severe AD and NPCAD compared with normal subjects (Fig. 1b). Moreover, the number of PV-positive cells was significantly reduced in the NBM of severe AD and NPCAD postmortem brains (Fig. 1c). Interestingly, the decrease in the PV level in NPCAD brains was similar to that of severe AD, which implies that the decrease of PV in the NBM of patients with AD occurs at the early stage.

To determine whether similar alterations occur in an experimental model of AD, we examined PV expression in the magnocellular preoptic nucleus (MCPO) of wild-type (WT) and 5×FAD mice. It is well known that the MCPO region of the rodent brain has cortical projection pathways similar to the NBM region of the human brain[50]. As expected, PV immunoreactivity signals were decreased in the MCPO of 5×FAD mouse brains compared with WT brains. Moreover, the density of PV was significantly decreased in the MCPO of 5×FAD mouse brains (Fig. 1d and e). Together, these results demonstrate that PV-expressing neurons in the BF are selectively reduced in both human AD brains and an AD mouse model, suggesting that PV neuron dysfunction in this region may represent an early pathological feature of AD.

### PV knockdown in the BF increased adjacent neuronal damage and impaired cognitive functions

Given the marked reduction of PV expression in the BF of AD brains, we next asked whether loss of BF-PV neurons contributes functionally to AD-related neuropathology and behavioral deficits. While the rapid-onset 5×FAD model was utilized to establish this baseline vulnerability to severe amyloid pathology, we transitioned to the more progressive APP/PS1 model for our subsequent *in vivo* manipulations. This approach provided a wider experimental window to chronically evaluate the long-term electrophysiological and behavioral consequences of targeted PV knockdown without the confounding effects of premature physiological decline.

**Figure 2:**
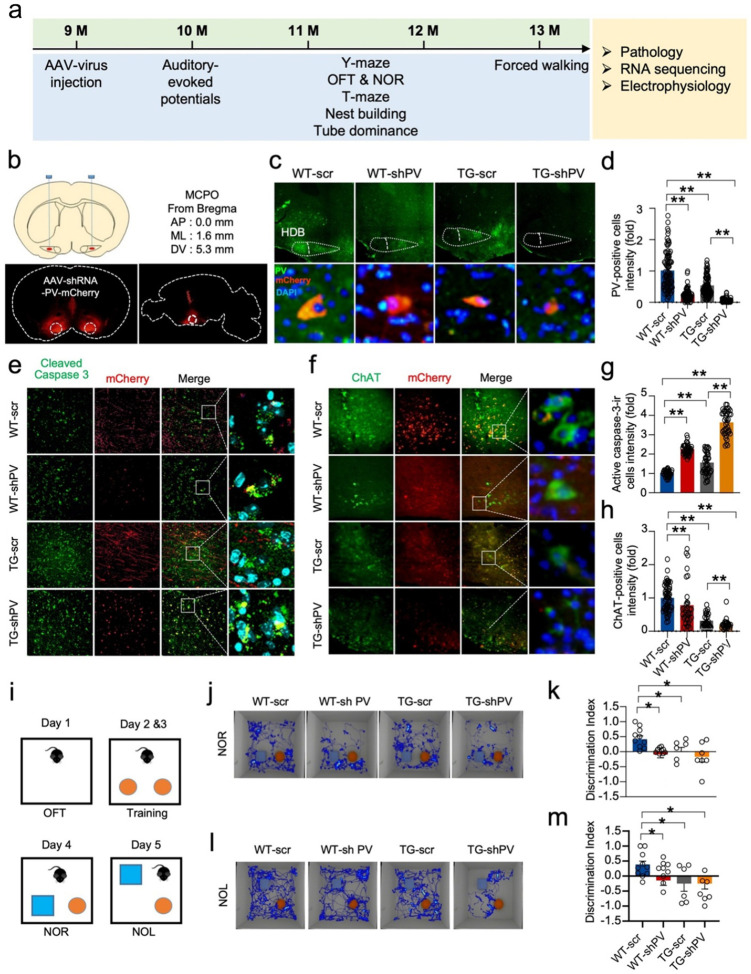
PV knockdown exacerbated the neuronal damages in the BF. **(a)** Schematic illustration of the experimental procedure used for EEG recordings, behavioral tests, and neuropathology assessments after PV knockdown. **(b)** Schematic illustration of the *in vivo* delivery of the AAV-shRNA-PV-mCherry virus to the MCPO of WT and TG (APP/PS1) mice. Detection of the mCherry signal confirmed that the virus was delivered to the MCPO of WT mice. (**c and d**) PV immunoreactivity was decreased in the MCPO of the brains from TG mice compared with WT mice. PV knockdown by shRNA of PV further reduced PV immunoreactivity in both the WT-shPV and TG-shPV groups. A total of 30 cells was counted (10 cells per mouse) in each group of mice (N =3). **(e and g**) The intensity of cleaved caspase-3 was significantly increased in mCherry-positive and adjacent neurons after PV knockdown. A total of 30 cells was counted (10 cells per mouse) in each group of mice (N =3). **(f and h)** ChAT immunoreactivity was decreased in the MCPO of TG mouse brains compared with WT mice. PV knockdown by shRNA of PV further led to a decrease in ChAT immunoreactivity in both the WT-shPV and TG-shPV groups. A total of 30 cells was counted (10 cells per mouse) in each group of mice (N =3). **(i)** Schematic diagram of the novel object tests. **(j and k)** In the novel object recognition (NOR) test, PV knockdown in both WT and TG mice resulted in significant changes in the discrimination index compared with the control (scr; scrambled shRNA) groups (WT-scr, N = 10; WT-shPV, N = 10; TG-scr, N = 6; TG-shPV, N = 7). Moreover, the discrimination index was significantly reduced in the TG-scr group compared with the WT-scr group. **(l and m)** In the novel object location (NOL) test, the discrimination index was significantly decreased after BF-PV knockdown in both WT and TG mice (WT-scr, N = 10; WT-shPV, N = 9; TG-scr, N = 6; TG-shPV, N = 7). Moreover, the discrimination index was significantly reduced in the TG-scr group compared with the WT-scr group. **(n)** Schematic diagram of the social dominance test. **(o)** In the social dominance test, PV knockdown in both WT and TG mice led to a lower winning rate compared with the control (scr) groups. Statistical significance was analyzed by two-way ANOVA followed by Tukey’s multiple-comparisons test. Data are presented as mean ± SEM. * P<0.05, ** P<0.01.

Accordingly, we generated AAV-shRNA-control and AAV-shRNA-PV constructs to determine whether *in vivo* loss of PV function in the BF affects the neuropathology, electroencephalography (EEG) features, and behaviors in mice (Fig. 2a). The experimental flows for EEG, behavioral tests, and neuropathology analysis after BF-PV downregulation are shown in Fig. 2a. We delivered AAV-shRNA-control or AAV-shRNA-PV viruses into the MCPO using the bilateral stereotaxic injection method (Fig. 2b). To confirm viral expression, we performed mCherry immunostaining on serial sections of WT mice. mCherry signals were found from AP 0.75 to −0.25 mm, based on bregma (**Supplementary Fig. 4**). mCherry expression was also detected in the medial septum (MS) region. This result indicates that PV-positive neurons in the MCPO project directly into the MS region. Knockdown of PV induced a loss of PV-positive neurons in the MCPO of WT mice and further exacerbated neuronal loss in TG mice (Fig. 2c and d).

To verify further whether PV knockdown causes neuropathological changes in the BF region, we examined the expression of cleaved caspase-3 and ChAT by immunofluorescence staining and confocal microscopy. Cleaved caspase-3 is a marker and indicator of apoptosis. In TG mice, the cleaved caspase-3 level was significantly elevated compared to WT mice. Moreover, the delivery of the AAV-shPV-mCherry virus enhanced the signal of cleaved caspase-3 in mCherry-positive neurons and neighboring cells in both WT and TG mice (Fig. 2e and g). Moreover, the number and immunoreactivity of ChAT-positive cells were markedly decreased in the BF of TG mice. Importantly, we noticed that knockdown of PV exacerbated the loss of ChAT-positive cells in the BF of WT and TG mice (Fig. 2f and h). Next, we examined the effect of PV knockdown in the BF on any cognitive functions by performing several hippocampal-dependent and prefrontal-dependent behavioral analyses. In the novel object recognition (NOR) and novel object location (NOL) tests (Fig. 2i), PV knockdown in both WT and TG mice resulted in significant changes in the discrimination index compared with the control (scr; scrambled shRNA) groups (Fig. 2j and l for NOR; and 2k and m for NOL). In the social dominance test, PV knockdown in both WT and TG mice yielded a lower winning rate compared with the control (scr) groups. TG with scrambled shRNA (TG-scr) led to a marginally lower winning compared with WT with scrambled shRNA (WT-scr) (P=0.051) in groups with the same genotype (Fig. 2n and o). In the open field test, PV knockdown in both WT and TG mice resulted in a significant decrease in center duration compared with the control (scr; scrambled shRNA) groups (WT-scr vs. WT-shPV, P<0.05; TG-scr vs. TG-shPV, P<0.05) (**Supplementary Fig. 5**).

### PV and ChAT neurons in the BF project to the MS and hippocampus

Because BF-PV knockdown produced pronounced cognitive and neuropathological alterations, we next investigated whether BF-PV neurons form direct neural circuits with brain regions involved in memory processing. To verify the network between PV neurons in the BF with those of other regions of the brain, we injected fluorogold (FG), which is a retrograde axonal tracer, into the MS and the dorsal and ventral hippocampus region, respectively. After the injections of FG, many neurons in the BF region were retrogradely and intensively labeled (**Supplementary Fig. 1a**). Microscopic images showed colocalization of ChAT (orange arrow)- or PV (yellow arrow)-positive cells with FG-labeled cells in the BF (**Supplementary Fig. 1a**). The number of FG-labeled cells in the BF was significantly higher from the retrograde projection of the MS than from the retrograde projection of hippocampal areas (**Supplementary Fig. 1b**). In addition, 35% of ChAT-positive and 5% of PV-positive cells in the BF region were labeled with FG from the retrograde projection of the MS (**Supplementary Fig. 1c, left panel**). Moreover, 31% of ChAT-positive and 15% of PV-positive cells in the BF region were labeled with FG from the retrograde projection of the dorsal hippocampus (**Supplementary Fig. 1c, middle panel**). Finally, 24% of ChAT-positive and 9% of PV-positive neurons were labeled with FG from the retrograde projection of the ventral hippocampus (**Supplementary Fig. 1c, right panel**). These findings indicate that BF PV neurons project directly or indirectly to the hippocampus, whereas ChAT neurons project extensively to both the median septum and hippocampal regions.

### BF-PV knockdown disrupted theta oscillations and theta–gamma coupling

**Figure 3:**
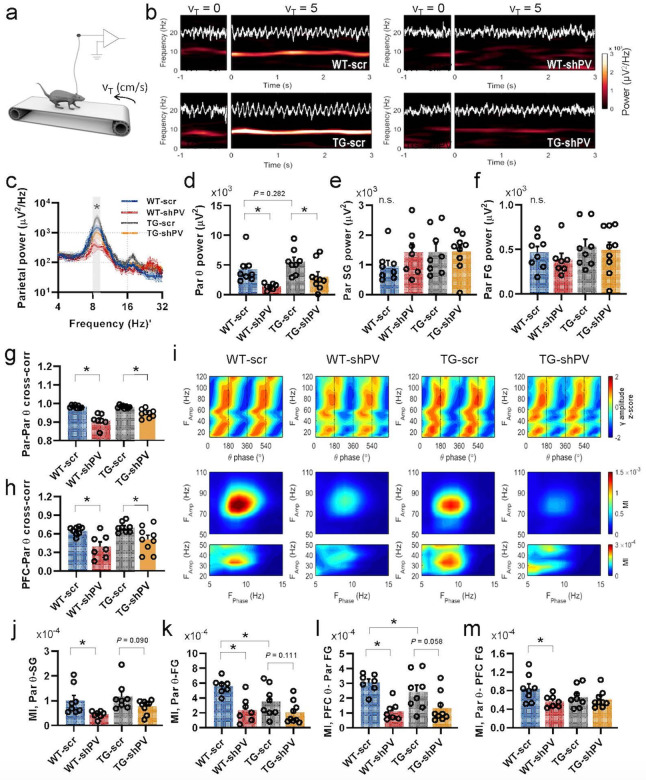
Disrupted theta oscillations and theta–gamma coupling after BF-PV knockdown were observed during treadmill walking. **(a)** Treadmill walking paradigm to induce steady locomotion and theta oscillation. **(b)** Representative spectrograms showing that a steady theta rhythm manifested after the control mice (scr) started to walk (speed, 5 cm/s), whereas this rhythm did not appear in the shPV groups. Par EEG traces (white) are overlaid on the spectrograms. The mice stayed on the treadmill at rest (from time −1 to 0 s) and walked on a running treadmill at a speed of 5 cm/s (from time 0 to 3 s). The polarity of the EEG electrodes was inverted to match the polarity of local field potentials. **(c)**Power spectrum obtained during the walking period. The gray shade indicates the frequency of significant differences in the shPV compared with the scr groups (8.0–9.5 Hz; *, P<0.05, Wilcoxon rank-sum test for each genotype). **(d)**The averaged Par theta power (6–12 Hz) was significantly reduced after knockdown of BF-PV in both WT and TG mice (WT-scr, N = 8; WT-shPV, N = 7; TG-scr, N = 8; TG-shPV, N = 9). **(e)** The averaged Par slow gamma power (SG, 20–50 Hz) was not significantly changed after knockdown of BF-PV in both WT and TG mice (WT-scr, N = 8; WT-shPV, N = 7; TG-scr, N = 8; TG-shPV, N = 9). **(f)** The averaged Par fast gamma power (FG, 60–100 Hz) was not significantly changed after knockdown of BF-PV in both WT and TG mice (WT-scr, N = 8; WT-shPV, N = 7; TG-scr, N = 8; TG-shPV, N = 9). **(g)**The cross-correlation between the parietal cortices in the theta band was significantly reduced in the shPV groups of both WT and TG mice (WT-scr, N = 8; WT-shPV, N = 7; TG-scr, N = 8; TG-shPV, N = 9). **(h)**The cross-correlation between the prefrontal and parietal cortices in the theta band was significantly reduced in the shPV groups of both WT and TG mice (WT-scr, N = 8; WT-shPV, N = 7; TG-scr, N = 8; TG-shPV, N = 9). **(i)**(Upper panel) The average amplitude of gamma oscillations with respect to theta phase (180° and 540° for troughs) showed that the SG and FG amplitudes became maximal at the falling phase and trough of theta oscillations, respectively; however, shPV mice (both WT and TG) exhibited disrupted phase–amplitude modulation patterns. (Lower panel) The averaged comodulograms showed disruption of phase–amplitude modulation after BF-PV knockdown in both WT and TG mice. Strong modulation patterns centered at 80 Hz (FG) and 35 Hz (SG) were obvious in the scrambled groups, but modulation became weaker in shPV mice (both WT and TG). **(j)** The modulation index of theta-SG indicated a significant decrease in modulation for WT-shPV mice compared with WT-scr, and a marginal reduction for TG-shPV compared with TG-scr (P=0.090) (WT-scr, N = 8; WT-shPV, N = 7; TG-scr, N = 8; TG-shPV, N = 9). **(k)**The modulation index of theta–FG revealed a significant decrease in modulation for WT-shPV mice compared with WT-scr mice, and a trend toward a reduction for TG-shPV compared with TG-scr mice (P=0.111) (WT-scr, N = 8; WT-shPV, N = 7; TG-scr, N = 8; TG-shPV, N = 9). **(l)** The modulation index of PFC theta–Par FG revealed a significant decrease in modulation for WT-shPV mice compared with WT-scr mice, and a marginal reduction for TG-shPV compared with TG-scr mice (P=0.058) (WT-scr, N = 8; WT-shPV, N = 7; TG-scr, N = 8; TG-shPV, N = 9). **(m)** The modulation index of PFC FG with respect to the Par theta phase decreased significantly in WT-shPV compared with WT-scr mice (WT-scr, N = 8; WT-shPV, N = 7; TG-scr, N = 8; TG-shPV, N = 9). Statistical significance was analyzed by two-way ANOVA followed by Tukey’s multiple-comparisons test. Data are presented as mean ± SEM. * P<0.05.

Given the anatomical connectivity between the BF and hippocampal circuitry, we next examined whether BF-PV dysfunction alters large-scale neural network activity. In this context, to investigate whether BF-PV knockdown affects cortical functions, mice were chronically implanted with EEG electrodes in the prefrontal cortex (PFC) and parietal cortex (Par). As shown previously, theta (6–12 Hz) and gamma (slow gamma (SG), 20–50 Hz; fast gamma (FG), 60–100 Hz) oscillations and their cross-frequency coupling (CFC) are required for encoding processes during memory formation[51–56]. Here, we examined theta and gamma dynamics during treadmill walking (Fig. 3a). As demonstrated previously[57], we found that Par EEG had prominent theta oscillations during treadmill walking. The representative spectrograms and overlaid traces of Par EEG depicted in Fig. 3b show that the scramble (scr) groups exhibited clear type 1 theta oscillations during active walking on a treadmill, whereas the shPV groups did not. Mouse locomotion appeared normal regardless of the genotypes and types of viruses injected. Power spectra recorded during treadmill walking (Fig. 3c) showed a dominant peak around 8.5 Hz, with a significant power difference observed between the scr and shPV groups in the theta band range for both WT and TG mice (7.5–11.3 Hz for WT-scr vs. WT-shPV; 8.0–9.3 Hz for TG-scr vs. TG-shPV). BF-PV knockdown triggered a reduction in theta power in both WT and TG mice (Fig. 3d), whereas its effect on gamma power was not significant (Fig. 3e–f). The cross-correlation between left and right parietal theta oscillations was significantly diminished in the shPV groups of both WT and TG mice (Fig. 3g). The frontal–parietal theta correlation was also reduced in both WT and TG mice after BF-PV knockdown (Fig. 3h).

The CFCs of theta phase and gamma amplitude were also disturbed in shPV mice. The average amplitude of gamma oscillations with respect to theta phase (Fig. 3i upper row; 180° and 540° for troughs) showed maximal SG and FG amplitudes at the falling phase and trough of theta oscillations, respectively, whereas shPV mice (both WT and TG) exhibited disrupted phase–amplitude modulation patterns. The averaged comodulograms presented in Fig. 3i (lower rows) showed that the strength of the phase–amplitude coupling was weaker in SG and FG bands for WT-shPV and TG-shPV mice compared with their scramble controls. The modulation index (MI) averaged over SG frequency ranges showed that BF-PV knockdown triggered a significant reduction in theta-SG coupling in WT-shPV and a trend toward a reduction in TG-shPV mice (Fig. 3j). Conversely, the averaged MI over FG frequency ranges revealed a significant difference between the WT-scr and TG-scr groups, as well as a reduced theta–FG coupling in the shPV groups (Fig. 3k). In addition to the disruption of theta oscillations in Par, the inter-regional communication in the theta band was also impaired after BF-PV knockdown: the CFC between PFC theta and Par FG was reduced significantly in WT-shPV and marginally in TG-shPV mice compared with the scramble groups (Fig. 3l). Moreover, the CFC between Par theta and PFC FG was also reduced in WT-shPV mice (Fig. 3m). The general properties of theta and CFC were preserved in baseline data, albeit with lower statistical power, which was summarized in a heatmap (**Supplementary Fig. 6**).

Next, we examined the correlations between the above-mentioned effects on EEG and behavioral dysfunction. In **Supplementary Fig. 2**, we report only correlations with P-values < 0.05 according to Pearson’s correlation coefficients. The winning rate in the social dominance test was significantly correlated with PFC theta power (r=0.66) and the inter-hemispheric similarity of Par theta oscillations (r=0.59). Similarly, the nesting score was significantly correlated with PFC theta power (r=0.54), and the time required for learning was significantly correlated with the inter-hemispheric similarity of Par theta oscillation (r=0.35 for spatial learning; r=0.73 for reversal learning). Interestingly, the theta peak frequency was significantly correlated with the nesting score (r=0.36) and the time required for spatial learning (r=0.60). In turn, the Par theta power was correlated with the latency in reversal learning (r=0.62). The alterations of PFC oscillations in treadmill walking and in evoked and steady-state responses are summarized in **Supplementary Figs. 7, 8 and 9**, respectively.

### BF-PV knockdown alters hippocampal transcriptomic signatures

**Figure 4:**
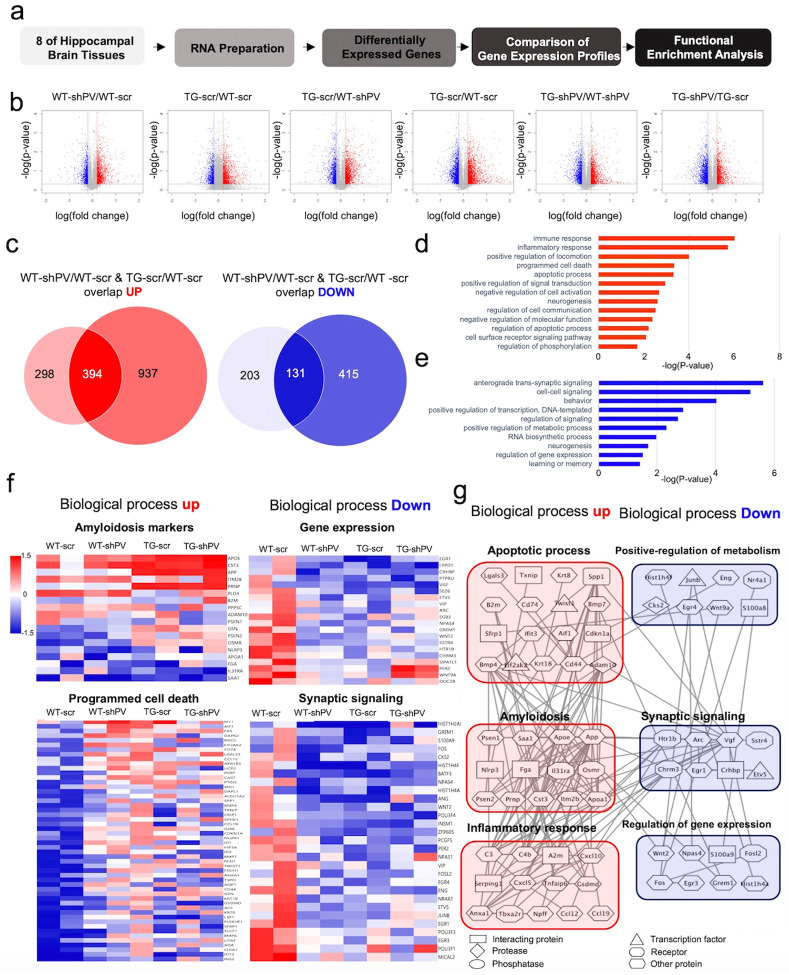
BF-PV knockdown decreased the expression of synaptic-transmission-related genes in the hippocampus. **(a)**Schematic illustration of the procedure fused in the hippocampal transcriptome analysis. **(b)** Volcano plots comparing Log2 (fold change) of Fragments Per Kilobase of transcript per Million mapped reads (FPKM) values for the indicated groups. Significantly upregulated genes are shown in red, and significantly downregulated genes are shown in blue. **(c)** The overlapped regions of Venn diagrams showed up- and downregulated gene levels in WT-shPV and TG-scr compared with WT-scr mice. **(d)** Top 5 KEGG pathway analysis of upregulated genes in both WT-shPV and TG-scr compared with WT-scr mice. Immune response was highly enriched in both WT-shPV and TG-scr compared with WT-scr mice. **(e)** Top 5 KEGG pathway analysis of downregulated genes in both WT-shPV and TG-scr compared with WT-scr mice. The synaptic signaling pathway was highly reduced in both WT-shPV and TG-scr compared with WT-scr mice. **(f)**Heatmap illustrating the up- and downregulated gene groups after PV knockdown in the BF of the hippocampus. Amyloidosis- and programmed-cell-death-related gene groups were elevated by PV knockdown in the BF. Gene expression- and synaptic-signaling-related gene groups were decreased after PV knockdown in the BF. **(g)** Network model showing that knockdown of PV in the BF was linked to a systematic deregulation of AD-related processes. The green nodes indicate genes with decreased mRNA expression levels, whereas the red nodes indicate genes with increased mRNA expression levels in both WT-shPV and TG-scr compared with WT-scr mice. The backgrounds represent the network modules for GOBPs, in which the epigenomes in the modules are involved. Node shapes represent the types of molecules, as indicated in the legend.

To understand better the molecular mechanism of disrupted theta oscillation and behaviors triggered by BF-PV knockdown, we conducted a transcriptome sequencing analysis using hippocampal samples. We collected a total of 8 hippocampal samples, 2 from each group. The procedure used in the hippocampal transcriptome analysis is illustrated in Fig. 4a. Using RNA sequencing, we obtained the average RNA sequencing throughput and 84.17% of reads aligned to the reference genome (Mouse mm10, UCSC Genome Browser). Volcano plots revealed that significantly upregulated and downregulated genes were well defined in each group of mice (Fig. 4b). Based on differentially expressed genes (DEGs) derived from each group, we identified the number of coMon and distinct DEGs in the WT-scr, WT-shPV, TG-scr, and TG-shPV groups (Fig. 4c). Among them, we focused on 394 upregulated and 131 downregulated overlapping genes between WT-scr and WT-shPV and between WT-scr and TG-scr mice, to investigate whether BF-PV knockdown yielded AD transcriptome signatures. A Kyoto Encyclopedia of Genes and Genomes (KEGG) pathway analysis of the top 5 upregulated genes in both WT-shPV and TG-scr compared with WT-scr mice revealed that immune response was highly enriched in both the WT-shPV and TG-scr compared with WT-scr groups (Fig. 4d). Moreover, a KEGG pathway analysis of the top 5 downregulated genes in both WT-shPV and TG-scr compared with WT-scr mice showed that synaptic signaling pathway was highly reduced in both the WT-shPV and TG-scr compared with WT-scr groups (Fig. 4e). Next, among the up- and downregulated pathways, we focused on amyloidosis, programmed cell death, gene expression, and synaptic signaling. A heatmap showed that BF-PV knockdown significantly affected the up- and downregulation of gene profiles in the hippocampus. Amyloidosis (*Apoe, App, Fga*, and *Psen2*)- and programmed cell death (*Aif1, Bmp4, Cast*, and *Fas*)-related gene signatures were elevated by BF-PV knockdown, whereas regulation of gene expression (*Egr1, Egr3, Egr4*, and *Fos*)- and synaptic signaling (*Arc* and *Chrm3* and *Wnt2*)-related gene signatures were decreased by BF-PV knockdown (Fig. 4f). Among the programmed cell death-related molecules, the *Anxa1, Cd44*, and *Mt1* genes were highly induced by BF-PV knockdown. To understand the functional associations of the BF-PV knockdown-landscaped transcriptomes in AD, we performed a biological network analysis (Fig. 4g). The biological network showed dense connections among the genes associated with amyloidosis, inflammatory responses, and apoptotic process, which were significantly changed in BF-PV knockdown mice and in AD (amyloid precursor protein (APP)/PS1) mice. (Fig. 4g). In particular, the downregulation of synaptic-signaling-pathway–related transcriptome signatures was strongly interconnected with the upregulation of amyloidosis and inflammatory-response–related transcriptome signatures. Amyloidosis (*Apoe, App, Fga*, and *Psen2*)-, apoptotic process (*Aif1, Adam10, Bmp4*, and *Cdkn1a*)-, and inflammatory response (*C3, C4b, Ccl5*, and *Cxcl10*)-related transcriptomes closely interacted between each group of genes involved in synaptic signaling (*Arc, Chrm3*, and *Sstr4*). These data indicate that the deregulation of BF-PV levels modulates the expression of several genes involved in essential cellular processes in the hippocampus.

### BF-PV knockdown enhances amyloidosis and reduces BDNF signaling in the hippocampus

**Figure 5:**
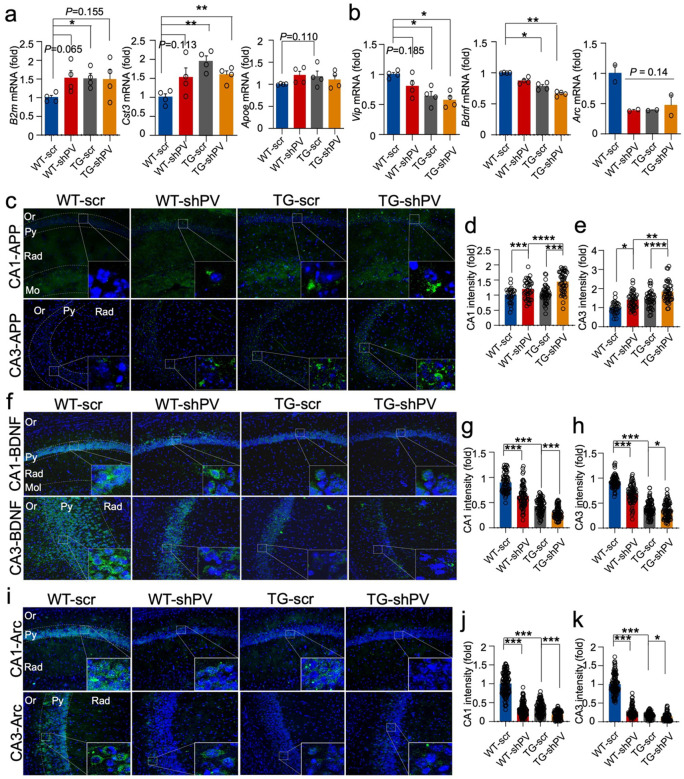
BF-PV knockdown exacerbated amyloidosis and reduced BDNF levels in the hippocampus. **(a)** PV knockdown by shRNA was associated with modest increases in *B2m, Cst3*, and *Apoe* expression, although these changes were not statistically significant. **(b)** Knockdown of PV by shRNA in the BF led to a decrease in the mRNA level of *Bdnf*, *Arc*, and *Vip* in the hippocampus (N = 4 per group). **(c–e)** The intensity of the amyloid precursor protein (APP) was significantly increased in the hippocampal CA1 and CA3 regions of 2×TG mice compared with WT mice. Knockdown of PV by shRNA in the BF exacerbated the expression of APP (10 cells per mouse; N = 3 mice per group). (**f**–**h**) The intensity of Bdnf was significantly decreased in the hippocampal CA1 and CA3 of 2×TG mice compared with WT mice. Knockdown of PV by shRNA in the BF exacerbated the loss of BDNF expression (10 cells per mouse; N = 3 mice per group). **(i–k)** The intensity of Arc was significantly decreased in the hippocampal CA1 and CA3 areas of 2×TG mice compared with WT mice (10 cells per mouse; N = 3 mice per group). The expression of Arc was further reduced by knockdown of PV in the BF. Statistical significance was analyzed by two-way ANOVA followed by Tukey’s multiple-comparisons test. Data are presented as mean ± SEM. * P<0.05, ** P<0.01, *** P<0.001.

To validate the change of transcriptomes at the molecular level, we next examined the expression of key AD-related and synaptic proteins in the hippocampus. As the hippocampus plays a major role in the formation of memory through a dynamic neuronal synaptic activity, we focused on determining how synaptic-activity-associated gene signatures are affected by BF-PV knockdown. To verify BF-PV knockdown-induced up- and downregulated genes, we performed qRT-PCR and found that the mRNA levels of the beta-2-microglobulin (*B2m*) and cystatin 3 (*Cst3*) genes were significantly increased in TG-scr and TG-shPV compared with WT-scr mice (Fig. 5a). Differences in *Apoe* mRNA levels were not detected among the groups (Fig. 5a). The vasoactive intestinal peptide (*Vip*), brain-derived neurotrophic factor (*Bdnf*), and activity-regulated cytoskeleton-associated protein (*Arc*) mRNA levels were significantly decreased in TG-scr and TG-shPV compared with WT-scr mice (Fig. 5b). In addition, to verify the cellular expression levels of the APP, BDNF, and Arc proteins, we performed immunofluorescence staining. The intensity of APP in the CA1 and CA3 was significantly increased in the TG-shPV compared with the WT-shPV and TG-scr groups (Fig. 5c–e). Concurrent with the qPCR results, the BDNF and Arc immunofluorescence intensities were significantly diminished in the CA1 and CA3 region in TG-scr and TG-shPV compared with WT-scr mice (Fig. 5f–k). These results indicate that BF-PV loss promotes amyloid pathology and suppresses activity-dependent synaptic signaling in the hippocampus.

### BF-PV knockdown shifts hippocampal synaptic balance toward inhibition

**Figure 6:**
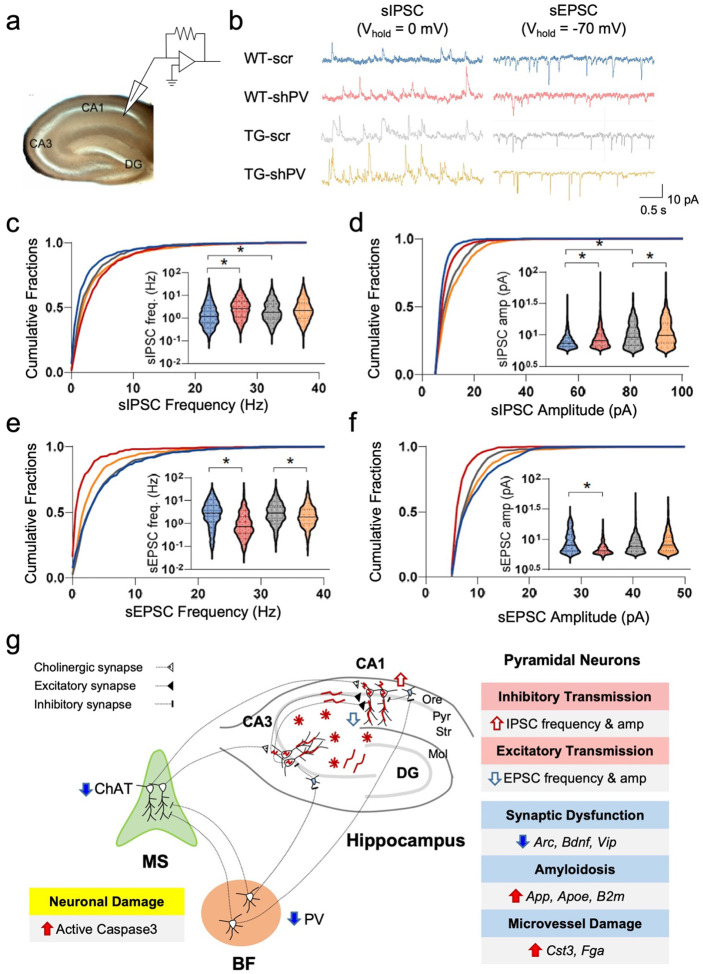
Gene silencing of PV in the BF alters the E/I balance toward inhibition in pyramidal neurons of the CA1 hippocampus. **(a)** Schematic diagram of whole-cell patch clamping of CA1 pyramidal neurons. **(b)** Representative traces of sIPSCs and sEPSCs. **(c, d)** Cumulative plot of the frequency and amplitude of sIPSCs across the groups. The insets show statistical differences across the groups. Gene silencing of PV in the BF increased the frequency and amplitude of sIPSCs, which was also observed in APP/PS1 transgenic mice (recording from 9 cells, N = 4 WT mice; 9 cells, N = 3 APP/PS1 mice). **(e, f)** Cumulative plot of the frequency and amplitude of sEPSCs across the groups. The insets show statistical differences across the groups. Gene silencing of PV in the BF reduced the frequency and amplitude of sEPSCs, which was not observed in APP/PS1 transgenic mice (recording from 8 cells, N = 4 WT mice; 9 cells, N = 3 APP/PS1 mice). **(g)** Schematic illustration showing that PV loss in the BF results in synaptic dysfunction, impaired neuronal transmission, and neuropathology in the hippocampus. Statistical significance was analyzed by two-way ANOVA followed by Tukey’s multiple-comparisons test. Data are presented as mean ± SEM. * P<0.05.

Because the above molecular changes by BF-PV knockdown suggested alterations in synaptic transmission and amyloidosis, we hypothesized that the alterations in the synaptic transmission of CA1 pyramidal neurons triggered by BF-PV knockdown are linked to changes in electrophysiological sequela. Accordingly, we performed a whole-cell patch-clamp analysis of CA1 pyramidal neurons to determine whether the spontaneous inhibitory and excitatory postsynaptic currents (sIPSCs and sEPSCs, respectively) were affected by BF-PV knockdown (Fig. 6a). The membrane potentials of the pyramidal neurons were held at 0 or −70 mV to record sIPSCs or sEPSCs, respectively (Fig. 6b). We found that BF-PV knockdown significantly increased both the frequency and amplitude of sIPSCs in WT mice (Fig. 6c–d), indicating the enhancement of inhibitory synaptic transmission onto CA1 pyramidal neurons. Enhancement of the frequency and amplitude of sIPSCs was also observed in TG mice, which agreed with the results of PV knockdown in WT mice. BF-PV knockdown in TG mice further increased the sIPSC amplitude, but not frequency, in the hippocampus. We also found that BF-PV knockdown significantly decreased both the frequency and amplitude of sEPSCs (Fig. 6e–f), indicating the suppression of excitatory synaptic transmission onto CA1 pyramidal neurons. Taken together, these results suggest that BF-PV knockdown alters the excitation/inhibition (E/I) balance toward inhibition in pyramidal neurons of the CA1 hippocampal area. This could be explained by the possibility that BF-PV-positive neurons could directly or indirectly project onto GABAergic interneurons in the hippocampus (Fig. 6g), as they directly target GABAergic interneurons in the neocortex^2,14^. Thus, the dysfunction of PV-positive neurons in the BF might cause disinhibition of hippocampal interneurons, thereby increasing inhibitory drive onto hippocampal pyramidal neurons.

### Multimodal analyses reveal cascade relationships between pathology, network activity, and behavior

**Figure 7:**
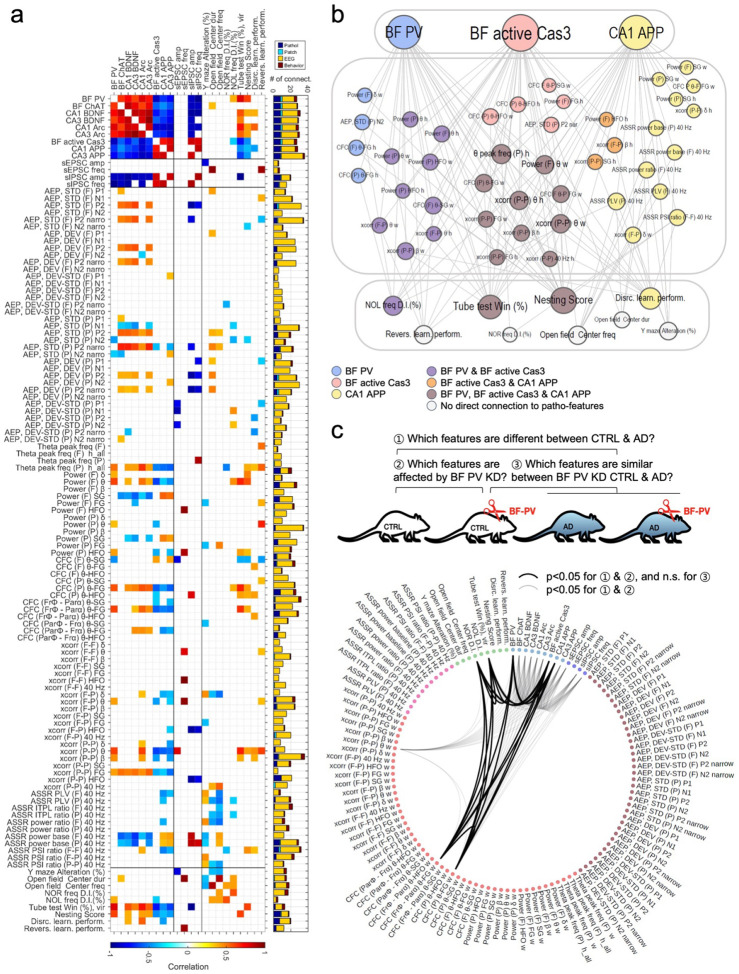
Statistical association between pathological, electrophysiological, and behavioral features. **(a)** (left) Heatmap of significant correlations between EEG features and behavioral and pathological features. The color denotes Spearman’s rank correlation coefficients with P<0.05 to reject the null hypothesis of no correlation. (right) Stacked bar chart indicating the number of significant connections. **(b)** Cross-modal correlation network for pathology, EEG findings, and behaviors. The pathological features of negative correlation with BF-PV were included in the network. The color of EEG/behavior nodes is a mixture of its nearest neighbors, indicating connectedness to the pathological features. The size of the nodes indicates the degree of connection. White nodes indicate features without significant correlation to pathological features. For simplicity, intra-modal connections and second-nearest EEG nodes were omitted. The network was visualized using the Gephi software. **(c)** Circulogram representing the similarity of statistical significance patterns. The nodes of features were linked if their statistical patterns agreed with the aimed questions depicted above the circulogram.

To understand how the diverse changes by BF-PV neuron are interconnected, we performed cross-modal correlation analyses across pathology, electrophysiology, and behavioral data sets. First, we performed a correlation analysis using all mice to identify triplet pairs having correlation between EEG and pathology, behavior and pathology, and EEG and behavior. Based on the enlarged dynamic ranges of the features, a substantial number of cross-modal pairs were determined to be statistically associated, as summarized in the correlation heatmap (Fig. 7a, P < 0.05, Spearman’s rank correlation). Overall, EEG variables related to effective neural synchrony were positively correlated with activity-dependent pathological features, such as PV, ChAT, Arc, and BDNF, in the hippocampus, and negatively correlated with neurodegenerative markers, such as APP and active Cas3. Similarly, the winning rate in the tube dominance test and nesting score were associated with the pathological data in a way similar to that observed for EEG. The triplets with significant associations across pathology, EEG, and behaviors are listed in **Supplementary Table 3** and visualized in a correlation network (Fig. 7b and **Supplementary Fig. 10**). In the correlation network depicted in Fig. 7b, the performance of discrimination in the T-maze was solely correlated with CA1 APP levels, whereas novel-location-recognition performance was correlated with BF-PV and active Cas3 levels. Percent win in the tube dominance test and nesting scores were correlated with all three pathological features. Moreover, some of the EEG features tended to be correlated with a single pathological feature, such as ASSRs with CA1 APP level, whereas other features, such as power, CFC, and regional correlation, were correlated with multiple pathological features. Behavioral features such as discrimination learning and reversal learning performance indicated the existence of a cascading effect after BF-PV loss, as they were not directly connected to BF-PV levels; rather, they were indirectly connected via the first-nearest neighbors of BF-PV. For simplicity, intra-modal connections (such as connections between EEG features) and second-nearest EEG nodes are omitted.

In particular, we analyzed the associations among pathological features. As was easily observable in the correlation heatmap of pathological data (upper panel in Fig. 7a), all pathological features were strongly associated with one another in the same direction among features with a similar effect on brain activity (see **Supplementary Fig. 11** for linear regression between pathological features). These omnipresent associations might be attributed to confounding factors; therefore, we applied partial correlation to assess independent relationships between two features (**Supplementary Fig. 3a**). BF-PV level showed a significant partial correlation with BF active Cas3 level when the other pathological features were controlled for (ρ=-0.62; P<0.05). As depicted in Supplementary Fig. 3b, the partial correlation analysis proposed a conjecture on the cascade association starting from BF-PV and extending to APP, BDNF, and Arc via BF active-cas3, and BF ChAT was located downstream in terms of association links. A cross-modal partial correlation analysis revealed more selective associations (**Supplementary Fig. 3c**).

To apprehend the interrelated group-level shifts in the features, we visualized a feature–feature circulogram based on their statistical patterns. For the set of critical questions (Fig. 7c, top), the nodes of features were connected if their statistical patterns agree with the aimed questions. The circulogram revealed that multimodal characteristics, such as BF-PV, CA1 APP, θ–FG coupling, and tube test win rates had analogous statistical patterns (Fig. 7c). These intermodal congruities of statistical patterns imply causal relations between them (direct or indirect).

### BF-PV as a key feature for ANN-based prediction of AD phenotypes

**Figure 8:**
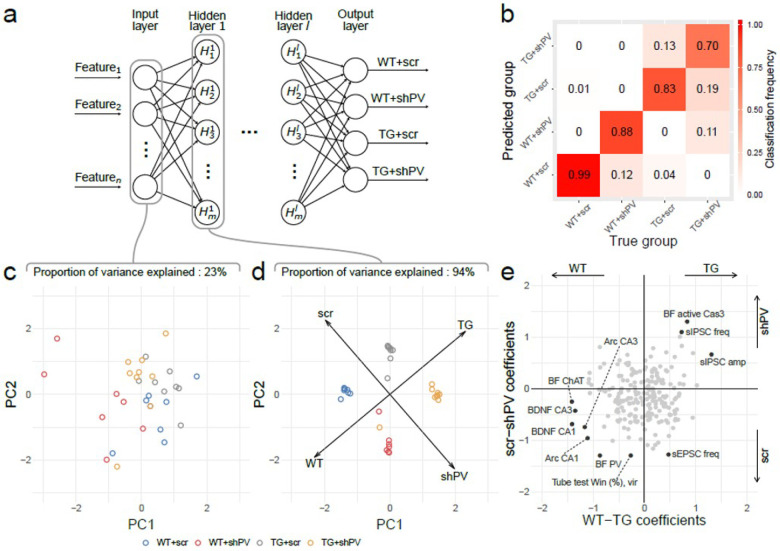
An artificial neural network (ANN) classifier uncovered that an AD transgene and BF-PV downregulation exert similar effects on the multimodal characteristics. **(a)** Schematic diagram of the MLP architecture. In the attribution analysis, l = 5 and m = 180 were used and the number of input features (n = 218). Each individual mouse was classified into a group that had the largest output layer activation. **(b)** Confusion matrix of the ANN classifier, with 85% accuracy. The colors and numbers denote the frequency at which the data from each group were classified into four possible scenarios**.(c, d)** PCA visualizations of ANN activations at the input layer and first hidden layer from an example model. The hidden-layer activations are predominantly confined in the 2-dimensional plane, with an explained variance ratio of 94%, which was consistent over the model ensemble. It also geometrically reflects the 2-by-2 (WT-TG and scr-shPV) data structure. The arrowed lines indicate the calculated WT-TG and scr-shPV unit vector. **(e)** WT-PV and scr-shPV attribution of input features. Black dots: high attribution features with top 5% norms. Gray dots: others.

The correlation analysis provided eminent evidence of the statistical association between BF-PV and AD phenotypes, but was not sufficient to establish the causation of the emergence of AD phenotypes by BF-PV knockdown. Therefore, we examined the causal association between BF-PV and AD by testing the significance of BF-PV for predicting the disease, i.e., its position as a deep feature for AD prediction. First, we built an artificial neural network (ANN) for group prediction with a multilayer perception (MLP, Fig. 8a) using 218 features from 32 mice. We performed a repeated 8-fold cross-validation accelerated by the ADAM optimizer, to optimize the architecture, and employed an ANN with 6 hidden layers and 180 hidden units for testing features (**Supplementary Fig. 11**). On average, the classification accuracies of the ANN were 84.5% for classifying four groups, 91.5% for three groups (WT-scr, WT-shPV, and TG), and 95.5% for two groups (WT-scr and the remaining groups) based on cross-validation (Fig. 8b). We further ascertained the ANN classification ability by comparing the discernibility between MLP layers using a principal component analysis (PCA). In hyperspace, the input layer activations were not converged into a low-dimensional principal plane (Fig. 8c), whereas the hidden-layer activations were converged to build four clusters that formed a near-orthogonal relationship with one another (Fig. 8d). The equidistant lines for two pairs of clusters divided the hyperspace into the quadrants to which each group belonged; therefore, we named each axis as the genotype and BF treatment axis, as depicted in Fig. 8d. Lastly, we determined the high attribution features that contributed to the performance of ANN using gradient-based attribution methods[47]. The attribution of each feature was calculated, and the features of the top 5% attributions were determined to be high attribution features (**Supplementary Fig. 12**). Among the pathological features, we found that PV, ChAT, and active Cas3 in the BF, and BDNF and Arc in the CA were critical for the performance of the ANN. In other data types, the winning rate in the tube test and sIPSC variables were features of high attribution. Most importantly, the distributions of each feature in the coefficient space showed that the directions of contribution were the same for TG and BF-PV knockdown and vice versa for WT and scr mice (Fig. 8e), implying that TG mice share multiple pathological, electrophysiological, and behavioral features with BF-PV knockdown mice. These results highlight BF-PV dysfunction as a central driver of hippocampal pathology, network dysregulation, and behavioral impairment in AD.

## DISCUSSION

In this study, we demonstrate that dysfunction of PV neurons in the basal BF disrupts hippocampal network activity, synaptic transmission, and cognitive function in AD models. Using a combination of human postmortem analyses, viral-mediated PV knockdown, electrophysiology, transcriptomics, and integrative computational approaches, we show that loss of BF-PV neurons triggers a cascade of molecular, circuit, and behavioral alterations. These findings identify BF-PV neurons as important regulators of hippocampal function and suggest that their dysfunction contributes to the pathogenesis of AD.

### Loss of BF-PV function and its association with pathological and behavioral abnormalities

Conventional views have suggested that dysfunction of BF cholinergic neurons is a major contributor to the cognitive impairment observed in patients with AD[30, 31]. Among BF subregions, the caudal portion—including the nucleus basalis (NB)—exhibits the most pronounced atrophy during early stages of AD compared with the rostral BF, which includes the medial septum (MS)[58]. The caudal BF, traditionally considered to project primarily to the isocortex, also sends substantial projections to the hippocampal formation and the isocortex, including the prefrontal cortex[1]. These projections suggest that the caudal BF may contribute to cognitive functions requiring coordinated activity between cortical and hippocampal networks, such as working memory.

In the present study, we observed that the number of PV-positive neurons was significantly reduced in the NBM of NPCAD and severe AD postmortem brains. Furthermore, PV immunoreactivity in the BF was significantly decreased in the 5×FAD mouse model of AD. To investigate the molecular mechanisms underlying the disrupted hippocampal theta oscillations and behavioral deficits induced by BF-PV knockdown, we performed transcriptomic analysis of hippocampal tissue. Notably, BF-PV knockdown increased the expression of genes associated with amyloid-related responses (*App, Fga*, and *Cst3*) and inflammatory responses (*C3, C4b*, and Cxcl10), while reducing the expression of genes associated with synaptic signaling and plasticity (*Arc, Bdnf, Vgf*, and *Chrm3*) in the hippocampus. These findings suggest that BF-PV dysfunction impairs neuronal synaptic activity and contributes to progressive synaptic deterioration associated with cognitive decline in AD. ARC and BDNF are well-established regulators of synaptic structure, plasticity, and memory formation, and their dysregulation has been widely reported in AD.

Behaviorally, deregulation of PV neurons in the BF of both WT and TG mice resulted in reduced exploration of novel objects in the NOR and NOL tests, indicating hippocampal dysfunction. The NOR test evaluates non-spatial object memory involving multiple brain regions, whereas the NOL test assesses spatial memory that depends primarily on hippocampal activity. These findings indicate that loss of BF-PV neuronal function disrupts neural circuits supporting hippocampal-dependent memory. A previous study by *Freund* and *Antal* demonstrated that the “*septohippocampal pathway*” plays an essential role in regulating hippocampal synaptic transmission[2]. Consistent with this concept, our electrophysiological recordings showed that BF-PV knockdown increased inhibitory synaptic transmission while reducing excitatory transmission in hippocampal CA1 pyramidal neurons. In addition, our neuropathological analyses revealed that BF-PV knockdown reduced the number of ChAT-positive neurons and decreased neurofilament intensity in the MS (**Supplementary Fig. 13**). These observations suggest that loss of BF-PV neurons leads to reduced cholinergic signaling within the BF–MS network and contributes to impaired amyloid metabolism and synaptic plasticity in the hippocampus. Collectively, the electrophysiological, EEG, neuropathological, and behavioral alterations observed following BF-PV knockdown suggest that loss of BF-PV function disrupts hippocampal synaptic transmission through the septohippocampal circuit[2, 11, 21, 25, 35].

### Impaired hippocampal theta oscillation and synaptic transmission by BF-PV downregulation

In the present study, we found that BF-PV knockdown caused significant alterations in rhythmic neuronal activity. Information processing in the brain relies on precise synchronization of neuronal ensembles, reflected in oscillatory power across frequency domains and phase–amplitude coupling between different rhythms[59]. In particular, theta–gamma coupling represents an effective mechanism for coordinating inter-regional communication and plays a crucial role in memory formation[60]. Although the specific rhythmic impairments vary depending on species and AD models, deficits in theta–gamma coupling have been consistently reported in AD animal models[55, 61] and in patients with AD, where they are associated with cognitive dysfunction[62]. Moreover, restoration of theta–gamma coupling has been observed following therapeutic interventions targeting AD-related network dysfunction[53]. Consistent with these observations, we found that theta–fast gamma (theta–FG) coupling was impaired in APP/PS1 mice, and that BF-PV knockdown in healthy mice produced an even greater reduction in theta–gamma coupling than that observed in APP/PS1 mice. This alteration was particularly pronounced in parietal EEG recordings, which are considered proxies of hippocampal activity. Previous studies have shown that reductions in theta–gamma coupling occur following focal knockdown in the hippocampus[63] or through systemic genetic knockout models[12]. To our knowledge, this is the first report demonstrating that focal knockdown of PV neurons in a remote brain region can disrupt theta–gamma coupling and impair cognitive processes.

Our pathological analyses revealed increased activation of cell death proteases in the BF and reduced expression of synapse-promoting proteins in the hippocampus. However, a direct mechanistic link between these molecular alterations and the reduction in theta–gamma coupling has not yet been established. One possible mechanism involves reduced synaptic inhibition onto PV-positive interneurons in the CA1 region. Fast synaptic inhibition plays a crucial role in shaping theta–gamma coupling[64]. In CA1, selective ablation of inhibitory inputs onto PV-positive interneurons reduces theta power and theta–gamma coupling without affecting gamma power[12], a pattern similar to the changes observed following BF-PV knockdown in our study. The mechanism by which BF-PV loss leads to reduced fast synaptic inhibition in the hippocampus is more difficult to determine. BF-PV neurons may provide an external source of inhibitory input to hippocampal PV interneurons either directly or indirectly through cholinergic pathways. However, several lines of evidence suggest that this effect is more likely mediated by direct projections rather than by cholinergic dysfunction. First, monosynaptic retrograde tracing studies have demonstrated direct projections from BF-PV neurons to CA regions[1], and other studies have shown that GABAergic projections from the BF, including those originating from PV neurons, selectively target hippocampal interneurons[65]. Second, our slice electrophysiological recordings in the CA1 region revealed changes opposite to those expected from cholinergic dysfunction. While BF-PV knockdown increased spontaneous inhibitory postsynaptic currents (sIPSCs) in CA1 pyramidal neurons, pharmacological blockade of cholinergic receptors in hippocampal slices has been reported to decrease sIPSCs[66]. In contrast, activation of cholinergic receptors increases sIPSC frequency in CA1 pyramidal neurons[67], further arguing against a cholinergic mechanism underlying the observed alterations in cross-frequency coupling. Nonetheless, several *in vivo* studies have reported that muscarinic acetylcholine receptor blockers reduce theta–gamma coupling in CA1[68] and in the medial entorhinal cortex[69]. However, unlike our observations, these manipulations also reduce gamma power, suggesting that cross-frequency coupling may have been reduced as a secondary consequence of decreased oscillatory activity. Given the complexity of mechanisms regulating theta–gamma coupling[70], further studies will be required to determine the precise origin of the reductions in theta power and theta–gamma coupling observed in BF-PV knockdown mice.

### Implications of BF-PV neuronal dysfunction in Alzheimer’s disease

In addition to established hypotheses of AD pathogenesis, such as cholinergic dysfunction and the amyloid cascade, our findings suggest an additional mechanism linking BF PV neuron loss to hippocampal network dysfunction. Previous studies have reported abnormalities in hippocampal PV interneuron activity in AD mouse models, including both excessive[71, 72] and diminished activity[73]. Age-related loss of PV neurons in PS1 knock-in mice and increased excitability of PV interneurons following amyloid-β exposure further suggest that hippocampal PV interneuron dysfunction is associated with amyloid pathology[71, 74]. However, several studies have indicated that hippocampal network oscillations may be disrupted even before significant amyloid-β accumulation occurs[54]. Interestingly, amyloid-β does not impair normal hippocampal circuits but can exacerbate memory deficits in circuits where PV interneurons are already hyperexcitable[72]. These observations suggest that the relationship between amyloid pathology and interneuron dysfunction is complex and may involve upstream circuit-level abnormalities.

Our findings raise the possibility that degeneration of BF-PV neurons contributes to dysfunction of the septohippocampal circuit, including impairment of ChAT-positive neurons, during AD progression. Although it is difficult to isolate the contribution of BF-PV neurons from other pathological factors in vivo, our results demonstrate that BF-PV loss initiates a cascade of molecular, electrophysiological, and behavioral changes resembling those observed in AD. Recent studies have also shown that stimulation of PV interneurons at gamma frequencies can reduce amyloid deposition[75] and rescue memory deficits in AD mouse models[53]. Given the critical role of PV neurons in generating gamma oscillations and regulating hippocampal network dynamics, it is plausible that BF-PV neurons contribute to AD pathology and cognitive impairment through their modulation of neural oscillations. Collectively, our findings provide new insights into the involvement of BF-PV neuronal dysfunction and associated neural circuits in the pathogenesis of AD.

### Conclusions

In conclusion, our study identifies BF-PV neurons as critical regulators of hippocampal network dynamics and synaptic function. Using human postmortem analyses, animal models, electrophysiological recordings, transcriptomic profiling, and integrative computational approaches, we demonstrate that loss of BF-PV neurons triggers a cascade of molecular, circuit, and behavioral abnormalities associated with AD. In particular, BF-PV dysfunction disrupts hippocampal theta–gamma coupling, alters synaptic transmission, and promotes transcriptional changes linked to amyloidosis and synaptic impairment. These findings reveal a previously underappreciated BF–hippocampal circuit mechanism contributing to AD pathogenesis. Targeting BF-PV neuronal circuits may therefore represent a promising strategy for restoring network function and mitigating cognitive decline in AD.

## Supplementary Material

The online version contains supplementary material available at…

This is a list of supplementary files associated with this preprint. Click to download.

- SupplemenatryInformation061326.docx

## Data Availability

The data that support the findings of this study are available from the corresponding author upon reasonable request.

## Abbreviations

AAV: Adeno-associated virus
ACSF: Artificial cerebrospinal fluid
AD: Alzheimer’s disease
AEP: Auditory evoked potential
ANN: Artificial neural network
APP: Amyloid precursor protein
APP/PS1: Amyloid precursor protein/presenilin 1
ASSR: Auditory steady-state response
BDNF: Brain-derived neurotrophic factor
BF: Basal forebrain
BF-PV: Basal forebrain parvalbumin
BUADRC: Boston University Alzheimer’s Disease Research Center
CFC: Cross-frequency coupling
ChAT: Choline acetyltransferase
DEGs: Differentially expressed genes
EEG: Electroencephalography
FITC: Fluorescein isothiocyanate
HFO: High-frequency oscillation
IHC: Immunohistochemistry
ITPL: Inter-trial phase-locking
KEGG: Kyoto Encyclopedia of Genes and Genomes
MCPO: Magnocellular preoptic nucleus
MI: Modulation index
MLP: Multilayer perceptron
MS: Medial septum
NACC: National Alzheimer’s Disease Coordinating Center
NBM: Nucleus basalis of Meynert
NEFM: Neurofilament medium
NOL: Novel object location
NOR: Novel object recognition test
NPCAD: Neuropathological and clinical Alzheimer’s disease
OFT: Open field test
PFC: Prefrontal cortex
PLV: Phase-locking value
PSI: Phase synchronization index
PCA: Principal component analysis
PBS: Phosphate-buffered saline
PFC: Prefrontal cortex
PV: Parvalbumin
qPCR: Quantitative real-time PCR
ROI: Region of interest
SEM: Standard error of the mean
sEPSC: Spontaneous excitatory postsynaptic current
sIPSC: Spontaneous inhibitory postsynaptic current
SG: Slow gamma
TBS: Tris-buffered saline
TG: Transgenic
UDS: Uniform Data Set
WT: Wild type
Xcorr: Cross-correlation coefficient
